# Identification of Serum Biomarkers for Intestinal Integrity in a Broiler Chicken Malabsorption Model

**DOI:** 10.3389/fvets.2019.00144

**Published:** 2019-05-09

**Authors:** Mikayla F. A. Baxter, Juan D. Latorre, Sami Dridi, Ruben Merino-Guzman, Xochitl Hernandez-Velasco, Billy M. Hargis, Guillermo Tellez-Isaias

**Affiliations:** ^1^Department of Poultry Science, University of Arkansas, Fayetteville, AR, United States; ^2^Departamento de Medicina y Zootecnia de Aves, Facultad de Medicina Veterinaria y Zootecnia, Universidad Nacional Autónoma de México, Mexico City, Mexico

**Keywords:** biomarkers, chickens, citrulline, IgA, intestinal integrity

## Abstract

Intestinal health is essential for feed efficiency and growth in animal agriculture and is dependent on barrier function, inflammation and dysbiosis. Our laboratory has published a nutritional model to induce gut inflammation using rye as a source of energy in poultry. More recently, we have used this model as an assessment of a nutritional rehabilitation model for better understanding of childhood undernutrition. The objective of this brief research report was to use a well-establish malabsorption model in broiler chickens using corn and rye as an energy source to identify several intestinal health biomarkers in the serum. To screen for inflammatory biomarkers, seven commercially available tests were used including Griess, superoxide dismutase, thiobarbituric acid reactive substances, Total antioxidant capacity, extracellular-signal-regulated kinase, Citrulline, and Interferon-ɤ; total IgA from cloacal swab was also measured. In the present study, chickens fed rye had a significant (*P* < 0.05) reduction in body weight and body weight gain at 10 day when compared with chickens that received the corn diet. In the second phase of the experiment, chickens that remain with the corn diet had significant differences in body weight and body weight gain. No significant differences were observed for any of the four antioxidant biomarkers evaluated in the sera (*P* > 0.05). However, significant differences were observed in serum citrulline and IFN-ɤ, as well as in cloacal IgA, in broiler chickens fed with rye, suggesting their potential use as biomarkers to study intestinal inflammation.

## Introduction

Intestinal health is essential for feed efficiency and growth in animal agriculture and is dependent on barrier function, inflammation, and dysbiosis (1, 2). Determining reliable, high throughout biomarkers to measure intestinal inflammation, and barrier function in poultry is continuously being investigated. In a recent study, Chen et al. (3) developed an inflammatory gut barrier failure model in chickens using high NSP (Non-starch polysaccharides) diet, and coccidiosis. Other biomarkers have also been evaluated to assess enterocyte health. Inflammation associated with oxidative stress can induce physiological changes in gene expression suggesting that inflammation-induced oxidative stress plays a crucial role in intestinal function (2, 3). Mitochondrial respiration is essential in maintaining TEER (Trans epithelial electrical resistance), suggesting oxidation plays an essential role in tight junction stability in Caco-2 cells (4). Decreases in mitochondrial ATP production resulted in a decrease in permeability and increase in the gene expression of occludin and claudin-1 but a decrease in the gene expression claudin-2 and claudin-7 providing a direct link between intestinal permeability, mitochondrial function, and cellular energy status (4). Oxidative stress is often measured by looking at metabolites produced during oxidation or enzyme activity. Superoxide dismutase (SOD) is an antioxidant enzyme that neutralizes the harmful by-products of metabolism (5). Thiobarbituric acid reactive substances (TBARS) are metabolites produced during peroxidation; total antioxidant capacity detects the antioxidant potential in a sample, and the Griess assay uses nitrite and nitrate breakdown to measure nitric oxide concentration within the cell (6). Our laboratory has published a nutritional model to induce gut inflammation using rye as a source of energy in poultry (3, 7–13). More recently, we have used this model as an assessment of a nutritional rehabilitation model for better understanding of childhood undernutrition (14). Citrulline is produced exclusively by the enterocytes of the small bowel, is the nitrogen end product of glutamine metabolism and can be converted to arginine (15). In pre-weaned piglets, plasma citrulline levels correlated with the intestinal absorption markers mannitol suggesting that citrulline can be used as a marker for intestinal function (16). The extracellular signal-regulated kinase (ERK) is a primary signaling pathway in the regulation of intestinal epithelial proliferation, survival, and wound healing (17). Therefore, it is plausible that ERK activity in the serum could reflect the intestinal damage incurred during stress.

On the other hand, secretory IgA (SIgA) is an essential part of the adaptive humoral immune system and the primary immunoglobulin that neutralizes pathogens on external mucosal surfaces while resisting proteases (18–20). LPS-induced TNF-α (Tumor necrosis factor-Alpha) is a transcription factor, which induces the expression of TNF-α, a pro-inflammatory cytokine (21). Interferon-gamma (IFN-ɤ), is another proinflammatory cytokine of the innate immune system with immunomodulatory and immuno-stimulatory properties (22–25). Hence, it is likely that both, innate and adaptive immune response, may provide a viable biomarker to assess intestinal health. The objective of this brief research report was to use a malabsorption model in broiler chickens to evaluate several intestinal health biomarkers in the serum and total IgA from gut mucosa.

## Materials and Methods

### Animals and Experimental Design

All animals and animal procedures were approved by the Institutional Animal Care and Use Committee at the University of Arkansas in Fayetteville. One hundred and sixty day old mixed sex broiler chicks from Cobb-Vantress, Silom Springs, AR, USA (*n* = 40 chickens/group) were used in this experiment. On the day of hatch, chicks were neck tagged and randomly allocated to one of four dietary treatments on group floor pens in temperature control rooms which were set using standard management practices. All diets were antibiotic-free and formulated to meet or exceed the current broiler nutritional requirements according to the National Research Council (1994; Table 1). The dietary treatments were (1) a control corn-based diet that chick consumed throughout the trial (corn-corn); (2) an early phase malnutrition diet where chicks were fed a rye-based diet during the first phase of the experiment and then in the second phase were fed a corn-based diet (rye-corn); (3) a control rye-based diet that chick consumed throughout the trial (rye-rye); (4) a late phase malnutrition diet where chicks were fed a corn-based diet during the first phase of the experiment than in the second phase were fed a rye-based diet (corn-rye). The experiment was divided into two phases, the first phase was from the day of hatch to day 10 and the second phase was from day 10 to day 20. At the end of the first phase, before the diets were switched, half the chickens were euthanized (*n* = 20 chicks/ diet). At the end of the second phase, the remaining chicks (*n* = 20 chicks/diets were euthanized to measure intestinal permeability after the diets were switched (corn-corn, rye-corn, rye-rye, corn-rye).

**Table 1:** Composition and nutrient content of the experimental diets (%).

**Item**	**Rye-based diet**	**Corn-based diet**
**Ingredients (%)**
Corn	–	57.32
Rye	58.27	–
Soybean meal	31.16	34.66
Poultry fat	6.30	3.45
Dicalcium phosphate	1.80	1.86
Calcium carbonate	1.10	0.99
Salt	0.38	0.38
DL-Methionine	0.35	0.33
Vitamin premix^a^	0.10	0.20
L-Lysine HCl	0.22	0.31
Choline chloride 60%	0.10	0.20
Mineral premix^b^	0.12	0.12
Threonine	0.08	0.16
Antioxidant^c^	0.02	0.02
**Calculated analysis**
Metabolizable energy (kcal/kg)	2850	3,035
Crude protein, %	22.38	22.16
Lysine, %	1.32	1.35
Methionine, %	0.64	0.64
Methionine + Cystine, %	0.98	0.99
Threonine, %	0.86	0.91
Tryptophan, %	0.30	0.28
Total calcium, %	0.90	0.9
Available phosphorus (%)	0.45	0.45
Sodium (%)	0.16	0.16

a *Vitamin premix supplied the following per kg: vitamin A, 20,000 IU; vitamin D3, 6,000 IU; vitamin E, 75 IU; vitamin K3, 6.0 mg; thiamine, 3.0 mg; riboflavin, 8.0 mg; pantothenic acid, 18 mg; niacin, 60 mg; pyridoxine, 5 mg; folic acid, 2 mg; biotin, 0.2 mg; cyanocobalamin, 16 μg; and ascorbic acid, 200 mg (Nutra Blend LLC, Neosho, MO 64850)*.

b *Mineral premix supplied the following per kg: manganese, 120 mg; zinc, 100 mg; iron, 120 mg; copper, 10 to 15 mg; iodine, 0.7 mg; selenium, 0.4 mg; and cobalt, 0.2 mg (Nutra Blend LLC, Neosho, MO 64850)*.

c *Ethoxyquin*.

### Assay Kits

To screen for inflammatory biomarkers, eight commercially available kits were purchased, and assays were conducted following the detailed description of the protocols for each assay, respectively. Oxidative stress kits were generic, and all kits were specific for chicken. The TBARS Assay kit, the SOD assay kit, and the antioxidant Assay kit were purchased from Cayman Chemical Company (Ann Arbor, Michigan, USA). The Griess reagent kit was purchased from Invitrogen™ Molecular Probes (Waltham, MA, USA) to determine nitrate concentration as an indicator of nitric oxide. The ERK and Citrulline sandwich ELISA kits were purchased from MyBioSource (San Diego, California, USA). IFN-ɤELISA kit was purchased from Invitrogen Corporation (Frederick, Maryland, USA). Total IgA concentrations were measured in the cloaca as previously described by Merino-Guzmán et al. (19).

### Statistical Analysis

All data were subjected to analysis of variance as a completely randomized design using the General Linear Models procedure of SAS (26). Data are expressed as mean ± standard error. Significant differences among the means were determined by using Duncan's multiple range test at *P* < 0.05.

## Results and Discussion

Inflammation is the endpoint of stress, regardless of its origin or nature (biological, environmental, nutritional, physical, chemical, or psychological). Stress and inflammation are innate responses involving hormones, immune cells, and molecular mediators, which are essential mechanisms for the survival and the healing process in all forms of life (20). During chronic inflammation, the increased production of reactive oxygen species induces peroxidation of lipids in cell membranes as well as mitochondria membranes (27). The long-term damage of this vital organelle has a profound impact on all cells of the individual. It is well accepted that in animals, the interactions between diet ingredients, gut microbiome, nervous system, immune system, and endocrine system play critical roles in metabolic and gastrointestinal disorders (28–30).

Evaluation of a nutritional rehabilitation model on body weight and body weight gain in broiler chickens fed rye or corn at varying time points are summarized in Table 2. In the present study, feed intake was not recorded. However, chickens fed rye had a significant (*P* < 0.05) reduction in body weight and body weight gain at 10 day when compared with chickens that received the corn diet. In the second phase of the experiment, chickens that remain with the corn diet had significant differences in body weight and body weight gain (Table 2). The reduction if body weight and body weight gain observed in the present study, are in agreement with previous studies conducted in our laboratory using a rye diet in modern broilers (11). In that study, a *Bacillus* direct fed microbial (DFM) was included in the same rye diet as a treatment group. Interestingly, although differences were observed in feed intake between control rye diet and DFM treated group, chickens that received the *Bacillus* spore based probiotic in the feed had a significant reduction in feed conversion ratio, digesta viscosity and liver bacterial translocation when compared with control non treated chickens (11). Similar results have been observed using other high non-starch polysaccharide diets (12, 13).

**Table 2:** Evaluation of a nutritional rehabilitation model on body weight and body weight gain in broiler chickens fed rye or corn at varying time points.

**Day**	**Treatment**	**Variable**
		**Body weight**
1	Corn	40.11 ± 0.33^a^
	Rye	39.85 ± 0.33^a^
10	Corn	175.91 ± 1.73^a^
	Rye	151.74 ± 1.76^b^
20	Corn-Corn	715.5 ± 5.84^a^
	Rye-Corn	695.85 ± 5.84^a^
	Rye-Rye	393.59 ± 6.34^c^
	Corn-Rye	453.8 ± 5.84^b^
		**Body weight gain**
1–10	Corn	135.80 ± 1.78^a^
	Rye	112.63 ± 1.79^b^
10–20	Corn-Corn	546.4 ± 6.80^a^
	Rye-Corn	534.40 ± 6.80^a^
	Rye-Rye	251.29 ± 7.37^b^
	Corn-Rye	278.50 ± 6.80^b^

*Data is expressed as the mean ±SE*.

*a-c Indicates significant differences between the treatments within the column at each time point (P < 0.05)*.

The oxidative stress has been related not only to the increased production of free radicals but also to changes to the scavenging capacity of antioxidant systems. To adapt to oxidative stress, the antioxidant systems in the body contain antioxidant enzymes such as SOD and Glutathione peroxidase which are employed to protect the body from oxidative stress (31). Dietary supplementation of several nutraceuticals have been shown to markedly ameliorate oxidative damage induced by heat stress in broiler chickens (32, 33). Furthermore, serum biochemical biomarkers such as glucose, proteins, calcium, phosphorus, and alkaline phosphatase concentrations have been reported to evaluate seasonal variations between male and female broiler chickens (34). Similar biomarkers have also been used to evaluate the antioxidant and immune modulatory properties of dietary supplements (35–37).

At 10 days of age, only three biomarkers were evaluated (SOD, IgA, and Citrulline). No statistical differences (*P* > 0.05) were observed in any of these biomarkers between corn or rye treated chickens (data not shown). Similarly, at day 20, no differences were detected for any of the four antioxidant biomarkers in the sera (Table 3). These metabolites may be processed in the liver preventing them from being detected in the sera. Previous research used commercially available kits to detect the oxidative stress differences in the plasma between thermoneutral and acute heat stress-treated chickens. Acute heat stress significantly increased plasma TBARS but had no significant effect on SOD and ferric/reducing antioxidant power (38). Other studies have also shown that systemic increases in oxidative stress markers are likely dependent on the type of stress (39, 40).

**Table 3:** Evaluating biomarkers in the sera of broiler chickens fed rye and corn at 20 days of age^*^.

	**Griess (μM)**	**Superoxide dismutase activity (U/ml)**	**Thiobarbituric acid reactive substances (μM MDA)**	**Total antioxidant capacity (ng/mL)**
**Antioxidant biomarkers**				
Corn-corn	0.78 ± 0.10	6.99 ± 0.10	0.29 ± 0.04	23.5 ± 1.07
Rye-corn	0.79 ± 0.14	6.733± 0.40	0.48 ± 0.07	22.3 ± 1.50
Rye-rye	0.74 ± 0.13	7.63 ± 0.22	0.35 ± 0.12	23.6± 1.26
Corn-rye	0.82 ± 0.08	6.63 ± 0.38	0.32 ± 0.04	21.6 ± 1.40
**Enterocyte biomarkers**				
	**Extracellular signal-regulated kinase (ng/mL)**		**Citrulline (ng/mL)**	
Corn-corn	0.26 ± 0.06		0.09 ± 0.07^b^	
Rye-corn	0.20 ± 0.05		3.71 ± 1.79^ab^	
Rye-rye	0.25 ± 0.06		6.67 ± 3.60^a^	
Corn-rye	0.35 ± 0.06		6.70 ± 1.05^a^	
**Immune biomarkers**				
	**IgA (ng/mL)**		**IFN-**ɤ**(pg/ml)**	
Corn-corn	1171.1 ± 58.8^b^		133.8 ± 75.9^b^	
Rye-corn	1062.9 ± 102.5^b^		98.1± 34.3^b^	
Rye-rye	1982.9 ± 68.4^a^		951.4 ± 399.7^a^	
Corn-rye	1768.7 ± 106.7^a^		126.8 ± 33.2^b^	

* *Data are expressed as the mean ± SE*.

a, b *Indicate significant differences between treatments within each column, P < 0.05*.

While no significant differences were observed on ERK between treated groups, a significant concentration in serum citrulline was observed in chickens fed rye-rye or chickens fed corn-rye (Table 3).

On the other hand, circulating citrulline is mainly produced by enterocytes of the small bowel. For this reason, plasma or serum citrulline concentration has been proposed as a biomarker of remnant small bowel mass and function (41). Hence, in humans decreased level of plasma citrulline correlated with the reduced enterocyte mass independently of nutritional and inflammatory status (42, 43). Intestinal citrulline production originates mainly from the proximal small bowel, and probably from the middle and upper parts of intestinal villi. The primary precursor is glutamine, and derived amino acids, either circulating or exogenous (44). Glutamine, arginine, and possibly other amino acids such as proline or ornithine, also contribute to the intestinal production of citrulline (45).

Interestingly, using the same nutritional rehabilitation model from the present study, Baxter et al. (14), demonstrated that duodenum of modern broilers fed corn in the second phase of the experiment had statistically lower villi height (VH) and shorter crypt depth than rye-fed chicks. Previous research has found that the chicks consuming a high fiber diet had a higher epithelial turnover and an increase in VH, to try to compensate for the poor digestibility (46–48). Therefore, the increasing concentration of serum citrulline observed in chickens that received the rye diets correlates with our previous findings that rye increases duodenal surface area. Hence, our results confirm that serum citrulline is correlated to small bowel length as has been demonstrated in mammals (43–45).

The intestinal mucosa releases anti-microbial proteins and IgA. IgA is an antibody isotype specialized in protecting the intestinal mucosa, as well as inhibiting inflammatory processes, neutralizing bacterial toxins, and enhancing nonspecific defense mechanisms (e.g., lactoperoxidase and lactoferrin) (19, 49). Secretory IgA provides the first line of defense by preventing pathogen entry into the mucosa (50–52). Chicks fed rye in the second phase of the experiment had significantly higher levels of cloacal IgA than those fed a corn-based diet (Table 3). In human medicine, fecal secretory IgA is measured as an indicator of intestinal infection, celiac disease, and food allergies (44, 53, 54). In chickens, *Salmonella enterica* infection and treatment with a probiotic *Enterococci faecium* resulted in an increase SIgA in an intestinal wash compared to the control (43).

Furthermore, a rye-based diet has been associated with pathogenic bacteria, specifically *Clostridium perfringens* (3, 9–11). Therefore, the higher abundance of cloacal IgA in the chicks fed rye in the second phase of the experiment is likely due to the dysbacteriosis, intestinal inflammation, and gut permeability associated with rye diets in poultry (9, 10). The rye-rye treatment group also had a significantly higher amount of serum IFN-ɤcompared to any of the other dietary treatment groups (Table 3). This finding suggests that a rye-based diet increased the amount of systemic pro-inflammatory cytokine IFN-ɤ. Pro-inflammatory cytokines like IFN-ɤhave also been reported to increase the expression of the pore-forming tight junction protein like claudin-2 and decrease expression of the pore-sealing claudin tight junctions like claudin-1, 3, 4, 5, and 8 (46, 55). Therefore, it is evident that broilers consuming a rye-based diet for 20 days increased the systemic level of IFN-ɤwhich likely contributed to an increase in intestinal permeability by modifying tight junction distribution within the intestinal tract as has been previously demonstrated (14). In summary, cloacal IgA, serum citrulline, and IFN-ɤmay be used as potential biomarkers to study intestinal inflammation in chickens.

## Author Contributions

MB, GT-I, and SD conceived and planned the study. JL and GT-I supervised all research. GT-I and BH analyzed and interpreted data. RM-G, BH, SD, and XH-V revised the first version of the manuscript. XH-V and GT-I were responsible for the final editing of the manuscript. All the authors approved the final version of the manuscript.

### Conflict of Interest Statement

The authors declare that the research was conducted in the absence of any commercial or financial relationships that could be construed as a potential conflict of interest.
